# Randomised controlled trial protocol (GRIP study): examining the effect of involvement of a general practitioner and home care oncology nurse after a cancer diagnosis on patient reported outcomes and healthcare utilization

**DOI:** 10.1186/s12885-018-4005-6

**Published:** 2018-02-05

**Authors:** I. A. A. Perfors, C. W. Helsper, E. A. Noteboom, E. van der Wall, N. J. de Wit, A. M. May

**Affiliations:** 1 Julius Centre for Health Sciences and Primary Care, University Medical Centre Utrecht, PO Box 85500, 3508 GA Utrecht, The Netherlands; 2 Internal Medicine and Oncology, University Medical Centre Utrecht, Utrecht, The Netherlands

**Keywords:** Primary care, Cancer, General practitioner, Homecare oncology nurse, Decision making, Follow-up, Patient satisfaction, Healthcare utilization

## Abstract

**Background:**

Due to the ageing population and improving diagnostics and treatments, the number of cancer patients and cancer survivors is increasing. Policymakers, patients and professionals advocate a transfer of (part of) cancer care from the hospital environment to the primary care setting, as this could stimulate personalized and integrated care, increase cost-effectiveness and would better meet the patients’ needs and expectations. The effects of structured active follow-up from primary care after cancer diagnosis have not been studied yet. Therefore the GRIP study aims to assess the effects of structured follow-up after a cancer diagnosis, by a primary care team including a general practitioner (GP) and a home care oncology nurse (HON), on satisfaction and healthcare utilization of patients treated with curative intent.

**Methods:**

We will conduct a multicentre, two-arm randomised controlled trial in The Netherlands. We plan to include 150 patients who will be treated with curative intent for either breast, lung, colorectal, gynaecologic cancer, or melanoma. Further inclusion criteria are: age 18 years and older, able to answer questionnaires in Dutch, GP agrees to participate and the possibility to include the patient before the start of treatment. All patients receive care as usual. The intervention arm will receive additional structured follow-up consisting of a GP consultation before onset of treatment to empower the patient for shared decision making with the specialist and a minimum of three contacts with the HON during and after treatment. Primary outcomes are: patient satisfaction with care at the level of specialist, GP and nurse and healthcare utilization. Secondary outcomes include: quality of life, employment status, patient empowerment, shared decision making, mental health and satisfaction with given information. Repeated questionnaires, filled in by the participants, will be assessed within the 1-year study period.

**Discussion:**

This randomised controlled trial will evaluate the effects of structured follow-up after a cancer diagnosis by a primary care team including a GP and HON, for patients undergoing treatment with curative intent. Results from the present study may provide the evidence needed to optimally rearrange responsibilities in cancer care delivery and consequently improve cancer care and patient related outcomes.

**Trial registration:**

Trial number: NTR5909.

## Background

Due to the ageing population and improvements in diagnosis and treatment, the number of cancer patients and cancer survivors is increasing [1, 2]. The WHO estimates a worldwide increase in cancer incidence, from 14.1 million new patients in 2012 to more than 20 million in 2025 [3]. In addition, survival is improving in the Netherlands, there will be an estimated increase of 57% in cancer survivors in 2020 [4].

In the near future, health care systems in several countries, such as The Netherlands, United Kingdom, Australia, USA and Canada, will face several challenges in fulfilling the needs and demands of this growing cancer patient population [2, 5]. In addition to the rising numbers of cancer patients, other changes concerning the cancer care path will challenge the healthcare system, such as the increased variety in treatment options [2, 5] [5], the increasing numbers of cancer patients with comorbidity resulting from aging [4, 6, 7] of the population and the increased urge for patient involvement in decision making and self-management [8, 9]. Consequently, there is a need to create a personalised cancer care continuum for each patient, based on individual preferences, medical profile and best fitting treatment options [6].

Traditionally, management of cancer is delivered by in-hospital specialists. In countries where the general practitioner (GP) is the gatekeeper in the care system, such as the Netherlands, the GP has a long-lasting personal relation with the patient, is up to date with the patients’ medical history and preferences, and is considered as a trusted health care advisor by most patients [10]. These typical features of the GP provide opportunities for improving continuous and personalised care for the growing population of cancer patients [11]. Therefore, patients, health care workers, governmental and professional organisations suggest a more prominent role of the GP in the guidance of patients during their cancer journey with a focus on empowerment, psychological and lifestyle support and follow-up care in the chronic disease stage [4, 6, 7, 11]. Even though a substantial role for primary care is advocated in the Netherlands and internationally, involvement of primary care in cancer care remains sporadic and unstructured [2, 4, 6, 11, 12].

At the same time, Dutch health care reports indicate that in 2020 the workload for GPs regarding care for patients with cancer will increase by about 66% within the Netherlands [4]. In order to divide this workload, policymakers suggest to involve the whole primary care spectrum, including GPs and primary care nurses [4, 6]. Beside keeping the workload acceptable, involving a primary care team may affect hospital care use. [13, 14]. Also, increased GP involvement was associated with higher patient satisfaction with care and treatment decision [15–18].

Scarce evidence suggests favourable effects of increased involvement of primary care in shared decision making and guidance during treatment, starting from diagnosis [13–18]. However, to our knowledge, the effectiveness of structured active follow-up by a primary care team starting from cancer diagnosis has not yet been published. Therefore, we designed the so called ‘GRIP study’. In this paper, we describe the design and methods of the GRIP study.

## Methods

### Aim

The randomised GRIP study primarily aims to evaluate the effects of structured follow-up from primary care on patient satisfaction and health care utilisation for cancer patients treated with curative intent. In addition, we assess the effects on quality of life, mental health, patient empowerment, shared decision making and employment status.

### Design

GRIP is a multi-centre, two-armed randomised controlled trial in the Netherlands.

### Study population

We aim to include 150 newly diagnosed cancer patients who are to be treated with curative intent for one of the following types of cancer: breast cancer, colorectal cancer, all types of gynaecologic cancer, lung cancer, or melanoma. We primarily intended to include prostate cancer, but our study was incompatible with ongoing psycho-social research in this patient population in the participating hospitals.

### Inclusion criteria

Patients are eligible for study participation, when they meet all of the following criteria:

- Newly diagnosed with one of the following types of cancer: breast cancer, colorectal cancer, all types of gynaecologic cancers, lung cancer or melanoma. Not being recurrent disease.
- Cancer therapy is initiated with curative intent (cancer staged I-III).
- Patient’s general practitioner agrees to participate in the GRIP-study.
- Patient is 18 years or older.
- Patients can be included before start of the cancer treatment.
- Sufficient mastery of the Dutch language or translator available during study.

### Exclusion criteria

A patient who meets any of the following criteria will be excluded from participation:

- Major psychiatric disease or personality disorders.
- Unable to fill in questionnaires.

Patients will be first screened for in- and exclusion criteria in the hospital by nurse (practitioners) or medical doctors and secondly by the researcher.

### Recruitment and allocation

To ensure reaching the required sample size of 150 patients, we involved all three major hospitals (one academic and two-non-academic) located in the greater urban region of Utrecht, the Netherlands, in the study. In addition, researchers will visit all sites biweekly to motivate the sites for inclusion of patients. All four GP cooperative care organisations in the region, together representing 300 GPs, and two home care organisations employing primary care oncology nurses, participate in the study. The GP cooperative care organisations inform their member GPs about the GRIP study, and the GPs can decline to collaborate by opt-out.

Eligible patients will be recruited in the hospital by the treating physician or oncology nurse after the patient is informed of his/her cancer diagnosis. After verbal consent, the treating physician or oncology nurse informs the research team, who contacts the patient by phone the (working)day after diagnosis. Written informed consent is obtained from all participants by the researcher.

The researcher will randomise the participants to intervention or usual care by using an computer operated electronic randomisation module, which is designed and maintained by the independent data management department of the UMC Utrecht. For randomisation, gender, date of birth, study number and site of inclusion of the patient need to be filled in on the website. Minimisation is applied to ensure balance between groups in treating hospital and cancer type. Due to the nature of the intervention, patients and health care providers are not blinded.

### Intervention

**Fig. 1:**
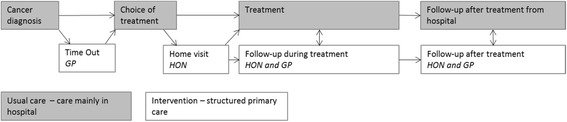
GRIP intervention in addition to usual cancer care. GP General Practitioner; HON Homecare Oncology Nurse

Patients in the intervention group are offered additional structured follow-up guidance from primary care, next to the usual secondary care, consisting of two components (Fig. 1):

1. A “Time Out consultation” with the GP between the moment of diagnosis and the final decision on treatment in secondary care.
2. Follow-up care from primary care, delivered by a home care oncology nurse (HON) in cooperation with the GP during and after active treatment. Active treatment includes surgery, chemo- and radiotherapy.

All components of the intervention are developed in close cooperation with the Dutch patient organisation “NFK (Dutch Federation of Cancer Patient Associations)” and the participating GP and home care organisations using existing healthcare services provided by regional organisations. Health care partners from the regional care network were chosen as preferred providers.

#### Time out consultation

After informed consent and before the final treatment decision are made in the hospital, the patient will be invited for an appointment with his/her GP for a ‘Time Out consultation’ of 20 min. In preparation, the GP of a patient randomised to the intervention group is contacted by the researcher to be informed about the intervention procedure. The researcher shortly explains the content of the “Time Out consultation” to the GP, including the topics of discussion with the patient as described below and an instruction to consult the HON after the Time Out consultation. In addition, the GP will be explained to not follow this structure when consulting cancer patients randomised to the control arm of the GRIP study in order to reduce contamination.

The Time Out consultation aims to facilitate continuity of primary care, to support the patient in a time of uncertainty, and to explore personal perspectives and preferences of the patients which may affect treatment choice to support shared decision making in secondary care.

During this consultation the GP addresses a number of issues preparing for active participation of the patient: reflection on the diagnosis and prognosis, psychosocial consequences, awareness that a choice of treatment exists and the recommendation to use the ‘three questions’ model in the consultation with the specialist on treatment decisions [19]. These three questions are: What are my options? What are the possible benefits and harms of those options? How likely are the benefits and harms of each option to occur in the patients’ specific information? Incorporating the three questions model in decision making has been demonstrated to improve the quality of information about therapeutic options and facilitate patient involvement [19].

#### Follow-up care during and after active treatment

After the Time Out consultation and the final treatment decision in secondary care, the Homecare Oncology Nurse (HON) will be contacted by the GP to schedule a visit at the patients’ home. During this visit the HON explains his/her role and makes a personal support plan together with the patient. In this plan, the patient’s situation is mapped on four domains: living conditions, physical, psychosocial and existential domain. If one of the domains requires active support, the HON discusses the required actions with the patient and with the GP.

The number, type and duration of contact moments with the HON is patient driven, with a minimum number of three contacts during the primary treatment phase, including the first home visit, and two contacts within 3 months after active treatment has ended. The content of contacts is based on the Dutch Distress Thermometer, which contains several items of the four domains on which patients are asked to rank their level of distress [20]. Throughout the cancer continuum the HON will report the status of the patient and the required actions to the GP, and if necessary the GP will be actively involved in the care provision. Secondary care will be actively approached by the HON, if supportive care, e.g., consultation of a psychologist, physiotherapist or dietician, is started based on HON’s consultations or when treatment-specific questions arise.

### Intervention training

All the participating HONs are registered nurses with a specialised training in oncology and have more than 2 years of clinical experience. In addition, the GRIP study team provides a 4-h training regarding supportive care, recognizing alarm-symptoms and the details of the GRIP intervention in order to be able to comply optimally with the intervention procedures. This includes close collaboration with the GP, the minimal content and frequency of consultations and the registrations required for the GRIP study. Expectations of all actors are displayed in Table 1.

**Table 1:** Expected actions for all actors to enable involvement of a primary care team after diagnosis

Patient	General Practitioner	HON
***Between cancer diagnosis and treatment***		
-Make appointment with GP for Time Out	-Prepare Time Out consultation -Execute Time Out -Contact HON for follow up during treatment	-Contacted by GP
***During and after treatment***		
-Contacted and visited by HON	-Informed on progress by HON	-Plans and performs patient contacts (proposed minimum two during and two after treatment.)
-Contact with GP if required	-Patient guidance if required	-Informs GP -If required consults GP/secondary care

*GP* General practitioner, *HON* Home care oncology nurse

Participating GPs receive basic information on the GRIP study by their GP cooperatives organisations at the start of the study. The GPs of patients who are randomised to the intervention group are notified by phone after the patient provides informed consent for participation. During this telephone contact, the researcher provides the necessary instruction to perform a Time Out consultation. In addition, information is given by e-mail and through a website which describes the steps GPs are expected to take. This website also provides the information required for optimal guidance from primary care and collaboration with HON and secondary care providers.

### Control group

Patients in the control group receive care as usual during the cancer journey. Hence for this group of participants, follow up guidance after diagnosis takes place in secondary care and guidance from primary care is not structured. Details of usual care depend on disease, patients- and caretaker characteristics, patients’ preferences and varying hospital protocols. In general, the phases of usual care can be described as: diagnosis, choice of treatment, delivery of treatment and follow-up care in hospital. Treatment options are discussed in a multidisciplinary team and generally follow national guidelines. Cancer care in the hospital is commonly delivered by a team consisting of a nurse (specialist) and a medical doctor specialised in oncology. In general, the GP is informed about the diagnosis by phone or by mail through Electronic Data Interchange after the multidisciplinary team reached consensus on the treatment.

### Outcomes

The primary outcomes are patient satisfaction with care and health care utilization. Secondary outcomes are health related quality of life, employment, patient empowerment, shared decision making, mental health and satisfaction with information.

### Primary outcome

To determine the primary outcome parameters the following validated questionnaires will be used: European Organisation for Research and Treatment of Cancer Satisfaction with care questionnaire (EORTC-IN-PATSAT32) [21], a Numeric Rating Scale (NRS) and the Medical Cost Questionnaire of the institute for Medical Technology Assessment (iMTA MCQ) [19, 22]. EORTC-IN-PATSAT32 consists of 32 questions and measures patients’ appraisal of hospital doctors and nurses, as well as aspects of care organisation and services [21]. The questionnaire will be adjusted to specify the satisfaction on specialists, GP and nurses. The NRS has a scale from 0 to 10 with the following question “How satisfied are you with the received care?”. Herein 0 implies “not satisfied at all” and a 10 implies that the patient “could not have been more satisfied” with the received care. The iMTA MCQ contains 31 questions and measures healthcare utilization (specific to the Dutch situation) [22]. The questionnaire will be adjusted to differentiate between the use of supportive care in primary or secondary care settings. Furthermore, questions evaluating medication use will be removed and questions evaluating the use of online websites and tools will be added. In addition, patients’ health records will be used to assess health care consumption.

### Secondary outcomes

The secondary outcomes are measured by eight questionnaires. Health related Quality of Life is assessed by the European Organisation Research and Treatment of Cancer-Quality of Life-C30 questionnaire (EORTC-QoL-C30), which incorporates functional scales (physical, role, emotional, cognitive and social functioning), one quality of life scale and symptom scales (including fatigue and pain) [23].

Employment is measured by the Productivity Cost Questionnaire of the institute for Medical Technology Assessment (iMTA PCQ), which contains 12 items [24]. Patient Empowerment will be measured based on two elements of empowerment, i.e. self-efficacy and Mastery. Self-efficacy is measured with the General Self-Efficacy Scale (GSE), a questionnaire with 10-hypothesises to assess optimistic self-beliefs to cope with a variety of difficult demands in life [25]. Mastery level will be measured with the Pearlin Mastery Scale, a 7-items questionnaire designed to measure self-concept and references the extent to which individuals perceive themselves in control of forces that significantly impact their lives [26]. Shared Decision Making will be measured using two questionnaires. The Shared Decision Making Questionnaire (SDM-Q-9) contains 9 items and assesses the effectiveness of interventions aimed at the implementation of SDM [27]. We added a question in order to evaluate the roll of the GP within this process, and the Perceived Efficacy in Patient-Physician Interactions (PEPPI), which contains 10 items [28]. Mental health is assessed by the RAND Mental Health Inventory (MHI-5), which contains 5 items and measures mental health [29]. Finally, satisfaction with information will be measured by the European Organisation for Research and Treatment of Cancer Assessment Satisfaction with information (EORTC-info 26), a 27-items cancer specific questionnaire which evaluates the information received by cancer patients [30]. In addition to the iMTA MCQ, several questions are added for the qualitative evaluation of online tools.

### Data collection

**Fig. 2:**
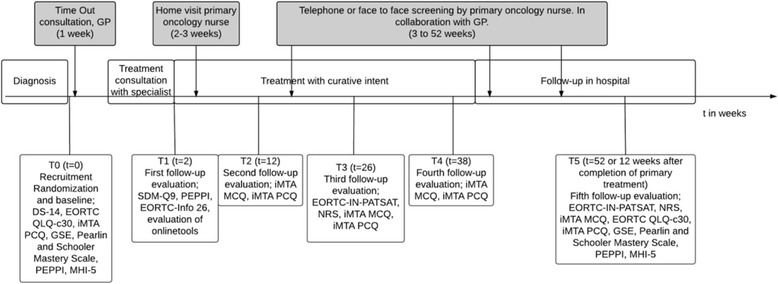
Questionnaire timeline in the cancer care pathway. GP General Practitioner; EORTC QLQ-c30 European Organisation for Research and Treatment of Cancer Quality of life Questionnaire; GSE General Self-Efficacy Scale; MHI-5 Mental Health Inventory; PEPPI Perceived Efficacy in Patient-Physician Interactions; SDM-Q9 Shared Decision Making Questionnaire; iMTA PCQ Productivity Cost Questionnaire of the institute for Medical Technology Assessment; DS-14 Assessment of negative affectivity, social inhibition, and Type D personality; EORTC-info 26 European Organisation for Research and Treatment of Cancer Assessment Satisfaction with information; iMTA MCQ Medical Cost Questionnaire of the institute for Medical Technology Assessment; EORTC-IN-PATSAT Satisfaction with care questionnaire; NRS Numeric Rating Scale

Data will be collected at baseline (T0), after 2 weeks (T1) and every 3 months (T2,T3,T4), up to 12 months after the diagnosis (T5) (Fig. 2). If primary treatment is already completed before T3 (e.g. for patients with a melanoma who only undergo surgery), patients receive the questionnaires from T3 directly and after 3 months the questionnaires of T5. The remaining questionnaires will be omitted. Questionnaires will be sent by email to the participant. When the participant does not fill in the questionnaires within 1 week, the electronic systems sends the participants one reminder. If this does not lead to completing the missing questionnaire, the researcher contacts the participant by phone for a final request to complete the questionnaire.

### Adherence

The researcher will register whether the Time Out consultation took place and the HON will register the number of contacts with the patient and the content of the contact moment by using a checklist. Participants can discontinue the study on request.

### Statistical analyses

All analysis will be performed following the intention-to-treat principle. Baseline characteristics will be shown by calculating means or medians for continuous variables and frequencies or percentages for categorical variables. Characteristics of patients who complete the study and patients who drop out, will be compared using T-tests for continuous variables and Pearson’s Chi-square analyses for categorical variables.

Linear regression analyses will be used for continuous variables adjusted for baseline variables (if measured at baseline) and treating hospital and cancer type. Mixed linear regression modelling adjusted for baseline variables as fixed factors (if measured at baseline) and stratification factors (treating hospital and cancer type) will be used to compare outcomes on repeated follow-up measurements T3 and T5. In these longitudinal analyses, the statistic model accounts for missing data based on the observed data [31]. Differential intervention effects due to sex (men/women), age (≤65/> 65 year), personality of type D (defined as 'scoring high on negative affectivity and social inhibition' [32]) (yes/no), type of cancer (breast/lung/colorectal/gynaecologic/melanoma), co-morbidity (none/1–2/> 3) and baseline levels of the outcomes of interest will be explored by adding interaction terms to the regression model.

### Sample size

We assumed a medium effect size (0.5) to be a clinically relevant difference in patients’ satisfaction between the two study groups. Using a power of 0.8 and an alpha less than 0.05, at least 64 patients per study group are required. Accounting for an estimated dropout of 15%, 75 participants in each group are needed.

## Discussion

The aim of the GRIP study is to assess the effects of structured follow-up from primary care after the diagnosis of cancer on satisfaction and healthcare utilization of patients treated with curative intent. To optimise personalised cancer care for a growing patient population and for effective implementation of structured follow-up from primary care, policymakers and professionals need more information on the effects of structured and continuous primary care involvement in the cancer continuum. The GRIP study will provide evidence on the effects on patient satisfaction and healthcare utilization and secondary outcomes. In this pragmatic study, patients with multiple cancer types will be included aiming at high generalizability of the results. It will be explored whether there are subgroups of patients for whom this structured primary care works best.

In this protocol some choices were made, that need clarification. First, we chose to assess the addition of structured follow-up from primary care by a GP and HON to care as usual instead of substitution of the supportive care provided in hospitals. This choice was made because we believe it is not feasible nor desirable to completely replace supportive care provided from secondary care by that from the primary care team. In addition, we aim to test the assumption that additional care from primary care will lead to a shift of the utilised care from the secondary to the primary care setting.

Second, patient satisfaction and healthcare utilisation are chosen as primary outcomes, since these factors are considered most relevant from the perspective of patient and society. Third, for the secondary outcome ‘patient empowerment’, so far, no uniform definition and no unique measurement tools exist. Therefore, we chose to use two validated questionnaires (GSE and Pearlin Mastery Scale) to estimate the effect of our intervention on patient empowerment. Although in previous intervention studies during cancer treatment comparable numbers of questionnaires were acceptable, the use of several questionnaires might induce loss to follow-up.

Last, we had to choose between random assignment at the patient or the caregiver level. We chose to randomise on patient level, using type of cancer and hospital for weighed randomisation, to ensure optimal comparison of study arms. To minimize the chance of contamination, GPs are only personally informed about the study details after one of their patients is randomised to the intervention. Given the low incidence of cancer in general practice (about 3 new patients meeting our inclusion criteria annually in an average general practice), we accepted the low chance of contamination resulting from the situation were one GP will first have a patient who is randomised to the intervention arm, followed by a patient randomised to the control arm.

### Trial status

The recruitment of participants started in April 2015. Patient inclusion will be completed in the first half of 2017. Patient follow-up is completed 1 year after the last patient will have been included.

## Abbreviations

DS-14: Type D Scale-14
EORTC QLQ-c30: European Organisation for Research and Treatment of Cancer Quality of life Questionnaire
EORTC-info 26: European Organisation for Research and Treatment of Cancer Assessment Satisfaction with information
EORTC-IN-PATSAT: European Organisation for Research and Treatment of Cancer Satisfaction with care questionnaire
GP: General practitioner
GSE: General Self-Efficacy Scale
HON: Home care oncology nurse
iMTA MCQ: Medical cost questionnaire of the institute for medical technology assessment
iMTA PCQ: Productivity cost questionnaire of the institute for medical technology assessment
MHI-5: Mental health inventory
NRS: Numeric rating scale
PEPPI: Perceived efficacy in patient-physician interactions
SDM-Q9: Shared decision making questionnaire
